# Biological Diversity in the Patent System

**DOI:** 10.1371/journal.pone.0078737

**Published:** 2013-11-12

**Authors:** Paul Oldham, Stephen Hall, Oscar Forero

**Affiliations:** 1 ESRC Centre for Economic and Social Aspects of Genomics (Cesagen), Lancaster University, Lancaster, United Kingdom; 2 Institute of Advanced Studies, United Nations University, Yokohama, Japan; 3 One World Analytics, Lancaster, United Kingdom; 4 Centre for Development, Environment and Policy, SOAS, University of London, London, United Kingdom; University of Catania, Italy

## Abstract

Biological diversity in the patent system is an enduring focus of controversy but empirical analysis of the presence of biodiversity in the patent system has been limited. To address this problem we text mined 11 million patent documents for 6 million Latin species names from the *Global Names Index* (GNI) established by the Global Biodiversity Information Facility (GBIF) and Encyclopedia of Life (EOL). We identified 76,274 full Latin species names from 23,882 genera in 767,955 patent documents. 25,595 species appeared in the claims section of 136,880 patent documents. This reveals that human innovative activity involving biodiversity in the patent system focuses on approximately 4% of taxonomically described species and between 0.8–1% of predicted global species. In this article we identify the major features of the patent landscape for biological diversity by focusing on key areas including pharmaceuticals, neglected diseases, traditional medicines, genetic engineering, foods, biocides, marine genetic resources and Antarctica. We conclude that the narrow focus of human innovative activity and ownership of genetic resources is unlikely to be in the long term interest of humanity. We argue that a broader spectrum of biodiversity needs to be opened up to research and development based on the principles of equitable benefit-sharing, respect for the objectives of the Convention on Biological Diversity, human rights and ethics. Finally, we argue that alternative models of innovation, such as open source and commons models, are required to open up biodiversity for research that addresses actual and neglected areas of human need. The research aims to inform the implementation of the 2010 *Nagoya Protocol on Access to Genetic Resources and the Equitable Sharing of Benefits Arising from their Utilization* and international debates directed to the governance of genetic resources. Our research also aims to inform debates under the Intergovernmental Committee on Intellectual Property and Genetic Resources, Traditional Knowledge and Folklore at the World Intellectual Property Organization.

## Introduction

In the mid-1990s patent protection was extended to all areas of invention by the Agreement on Trade-Related Aspects of Intellectual Property Rights (TRIPS) under the World Trade Organization (WTO) [1]–[3]. As a result of the TRIPS Agreement biological organisms and their components were incorporated into the realm of international patent protection with limited exceptions for the purposes of protecting *ordre public* or morality including protecting health or preventing serious prejudice to the environment (Article 27.2) [4], [5].

A patent is a temporary grant of a monopoly on the right to make, use, offer for sale, or import, an invention in a country where the patent is in force [6]. Patents are typically granted for 20 years. During this period, patent holders enjoy exclusivity over the protected invention or may licence or transfer the invention to others [7], [8].

The modern patent system is global in nature and is supported by regional and international patent treaties, notably the Patent Cooperation Treaty, that extend the system to 146 countries [9]. The global scale and diversity of the modern patent system presents challenges in arriving at a balanced view of its strengths and weaknesses. Appreciating the strengths and weaknesses of the patent system requires interdisciplinary engagement with the economic, scientific, social, legal and policy aspects of this global system.

On the side of the strengths of the system, it is often argued that patent protection is vital to the ability of various industry sectors, notably the pharmaceutical and biotechnology sectors, to generate a return on costly investments incurred during research and development in the face of competition [6], [7], [10]–[15]. In return for this temporary period of protection patent holders are required to disclose new and useful inventions that become available to the wider public when the patent expires. Patent protection is also regarded as an important tool for promoting Foreign Direct Investment by providing legal security for companies that their intellectual property will be respected in foreign jurisdictions [15]–[20]. This in turn is associated with the promotion of technology and knowledge transfers or spillovers across frontiers [21]–[26]. On a strategic level, patent protection is important for governments seeking to realize a return on investments of public money in science and technology for the benefit of their national economies and to maintain a competitive position in the world economy [8], [14].

A major weakness of the system involves the wider impacts and implications of the proliferation of claims to a particular form of property for science and society. This weakness is highlighted by controversies about patent activity for genetic resources and traditional knowledge [5], [27]–[29]. Within the scientific community these controversies focus on the impacts of patent protection on the freedom, orientation and basic costs of research [30]–[33]. Particular attention has focused on the control of research tools and basic elements of biology such as genes and whole genomes [34]–[38].

The proliferation of property claims is associated with ‘patent thickets’ that have become a deterrent to basic and applied research in fields such as breast cancer [39]–[42]. The increasing ‘enclosure’ of biology using patents is seen as a threat to basic scientific research and the free sharing of knowledge through the public domain [43]–[45]. In response to this weakness a range of movements have emerged around the concepts of the commons, open source and global public goods dedicated to sharing knowledge and maintaining access to basic scientific information and research tools [46]–[52].

In contrast, civil society, religious and expert bodies have focused on a spectrum of ethical and human rights issues raised by patent protection. These concerns range from the morality of patenting genes and life forms, to patents involving embryonic stem cells and efforts to secure patent rights over synthetic organisms in the emerging field of synthetic biology [53]–[56], [61]. The implications of patent activity for the respect and promotion of the human rights of vulnerable populations such as indigenous peoples has also been an intense focus of attention. Indigenous peoples are a focus of research with respect to their distinct genetic heritage and also for their knowledge of the useful properties of plants and other organisms (traditional knowledge) [5], [57]–[60].

On a wider level, debates involving developing countries have focused on issues such as the impacts of patents on access to basic medicines, including HIV antiretroviral drugs, and a need for innovation to address neglected diseases [37], [62]–[67]. In agriculture debate focuses on the implications of the promotion of patent protected technologies in genetic engineering for livelihoods, food security and the conservation of biodiversity in developing countries [68]–[71].

Developing countries have also expressed concern about the problem of ‘biopiracy’ or misappropriation of biological resources and traditional knowledge that becomes the basis for applications for patent rights across a spectrum of fields including cosmetics, herbal medicines, pharmaceuticals and biotechnology [72]–[77]. In response to this problem in 2010 the United Nations Convention on Biological Diversity adopted *The Nagoya Protocol on Access to Genetic Resources and the Fair and Equitable Sharing of Benefits Arising from their Utilization* (hereafter the Nagoya Protocol). This new Protocol establishes that those seeking to conduct research and development on genetic resources and traditional knowledge from a particular country must:

- seek prior informed consent from the relevant government;
- seek prior informed consent from relevant indigenous and local communities, and;
- establish a benefit-sharing agreement on mutually agreed terms [78]–[81].

In addition to the Nagoya Protocol, the World Intellectual Property Organization (WIPO) has established an Intergovernmental Committee on Intellectual Property and Genetic Resources, Traditional Knowledge and Folklore [5], [82]. Member states of WIPO are presently negotiating the draft of what may become a new international treaty to address the intellectual property dimensions of genetic resources, traditional knowledge and folklore.

Debates on intellectual property and biodiversity extend across the United Nations system. In the case of the world’s major food and forage crops debate focuses on the International Treaty on Plant Genetic Resources for Food and Agriculture [71], [83], [84]. The World Health Organization has focused on access to medicines, criteria for the exchange of influenza viruses and neglected diseases [85]–[87]. In the case of marine environments the United Nations Convention on the Law of the Sea, under the United Nations General Assembly, is considering the creation of a new instrument on marine genetic resources outside national jurisdictions such as hydrothermal vents and the deep sea bed [88], [89]. Outside the United Nations system, the Antarctic Treaty Council has considered the emergence of biological prospecting for extreme organisms within its jurisdiction [90]–[92]. In the realm of trade, debate is concentrated around the Council for Trade-Related Aspects of Intellectual Property Rights (TRIPS Council) at the World Trade Organization [93]. For many critics of the patent system and observers, the TRIPS agreement is the source of many of the problems highlighted above. However, at the time of writing there is no consensus between governments on proposals for reforms [94], [95].

Three key principles are emerging in ongoing debates about the governance of genetic resources and traditional knowledge. The first principle is that those who provide genetic resources and traditional knowledge, such as developing countries or indigenous peoples and local communities, should give their prior informed consent. The second principle is equitable benefit-sharing as articulated in the third objective of the Convention on Biological Diversity. That is, those who provide genetic resources and traditional knowledge should share in the benefits of research and development in ways that are mutually agreed with researchers or other users. The third principle is that of promoting access to genetic resources for research and development. This is grounded in the view that it is in the long-term interest of humanity to promote access to genetic resources for research and development leading to new knowledge and useful products.

One problem in intergovernmental debates about the governance of genetic resources and intellectual property is a lack of empirical research on biodiversity within the patent system to inform these debates. We sought to address this problem using a combination of large scale text mining, High End Computing, the federation of online and offline databases and analytics software. Our primary objective is to provide sound empirical evidence to inform debates on intellectual property and biodiversity. However, the patent system also provides an important window into human innovations that utilize biodiversity. That is, it reflects dominant trends in human relationships with biodiversity through the medium of claims to property. In this article our aim is to map the major contours of patent activity involving biodiversity with a particular focus on subject areas of interest to specialists from a range of disciplines to encourage further research. We conclude by considering how the limitations of research and innovation involving biodiversity exposed through our research might be overcome.

## Methods

The research used High End Computing to text mine 11 million patent documents from the United States, the European Patent Convention and the international Patent Cooperation Treaty published between 1976–2010 for 6 million binomial Latin species names from the *Global Names Index* (GNI) established by the Global Biodiversity Information Facility (GBIF) and Encyclopedia of Life (EOL). The results were then cleaned, harmonized and federated with online taxonomic databases using web services from GBIF and the Species 2000 & ITIS *Catalogue of Life* and the offline *EPO World Patent Statistical Database* (PATSTAT, October 2011 edition). Data was manually validated and tested in Vantage Point text mining and analytics software from Search Technology Inc. and Word Smith corpus linguistics software with independent tests performed using samples from the commercial Thomson Innovation database. Visualization of federated data was performed in Tableau analytics software with network mapping performed in open source Gephi software. Details of the methods employed for the research are provided in the Supporting information S1 file. Workbook S1 provides the data discussed in this article as a contribution to further research.

## Results

**Figure 1:**
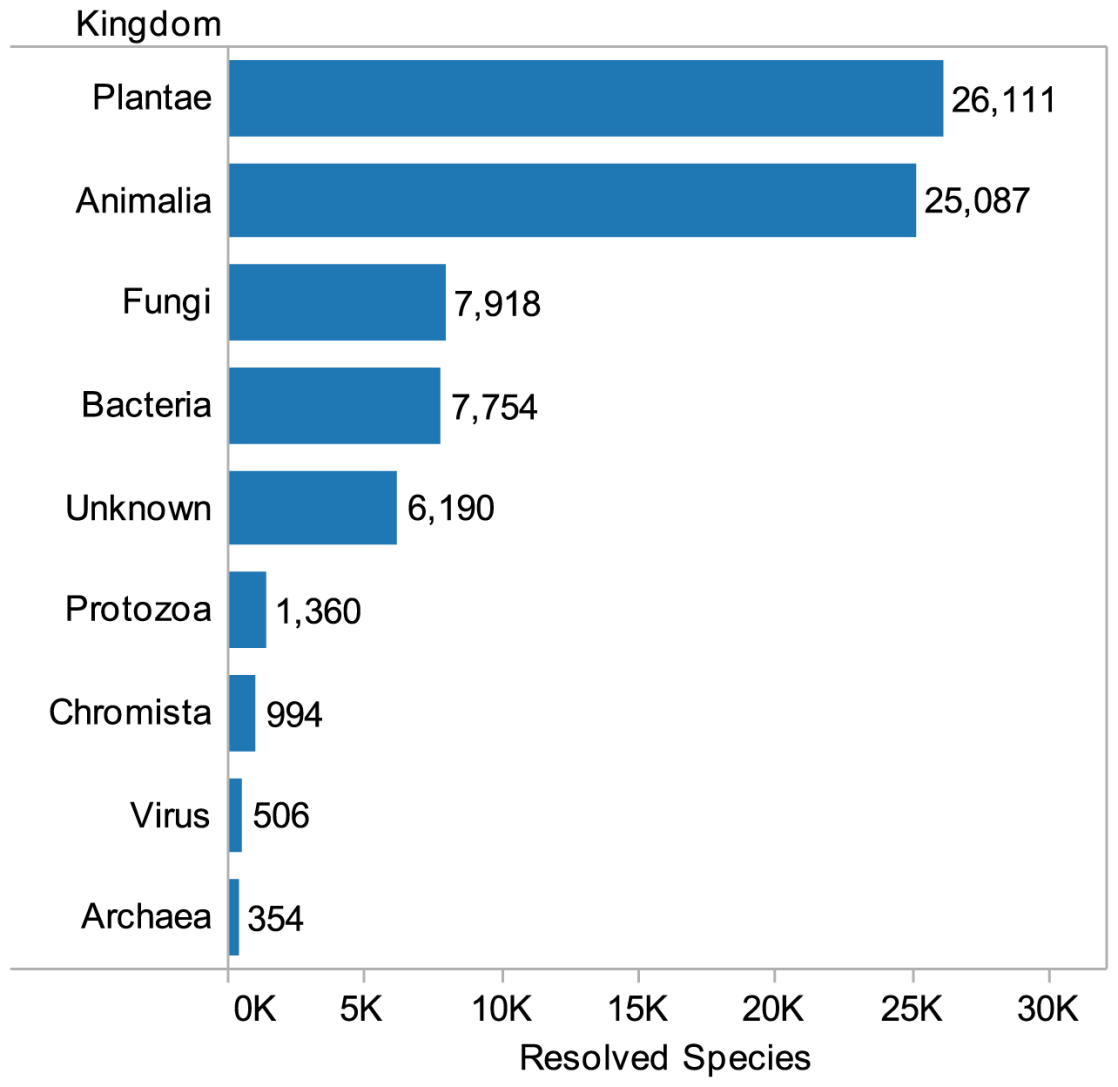
Species in Patents by Kingdom. Species appearing in patents grouped onto the major kingdoms based on the resolved species names in Workbook S1, table S2.1 excluding unresolved abbreviations.

Using the *Global Names Index* (GNI) we identified 95,303 raw Latin species names in the whole texts of 11 million patent documents from the United States, the European Patent Convention and the Patent Cooperation Treaty in the period 1976–2010 (Supporting information S1, Workbook S1, table S1) [96]. This data includes spelling variations, equivalent names, partial names, and 6,999 unresolved abbreviated names (Workbook S1, table S1). These results were resolved to 83,274 species names including unresolved abbreviations (Workbook S1, table S2). Following the removal of unresolved abbreviations we arrived at a total of 76,274 full Latin species names from 23,882 genera across the major kingdoms as summarized in Figure 1 (Workbook S1, table S2.1). Due to name variations and synonyms we regard the 76,274 species names as an upper estimate for the presence of biodiversity in the selected patent collections based on Latin species names. With the exception of major food crops we did not address common names for species. Due to the complexity of virus taxonomy we anticipate that viruses may be under-represented in the data.

1,347,224 million species are listed within the Species 2000/ICTIS *Catalogue of Life Annual Checklist 2011* representing up to two-thirds of approximately 1.9 million described species [97], [98]. Existing estimates for overall species numbers on Earth range from 3 to 100 million with recent research predicting a total of 8.7 million species (±1.3 million) [99]. This range of figures reveals the limitations of scientific knowledge of global biodiversity. However, these figures suggest that human innovative activity represented in the patent system focuses on approximately 4% of taxonomically described species (assuming 1.9 million described species) and a range between 0.8–1% of predicted global species (assuming 8.7 million ±1.3 million).

**Figure 2:**
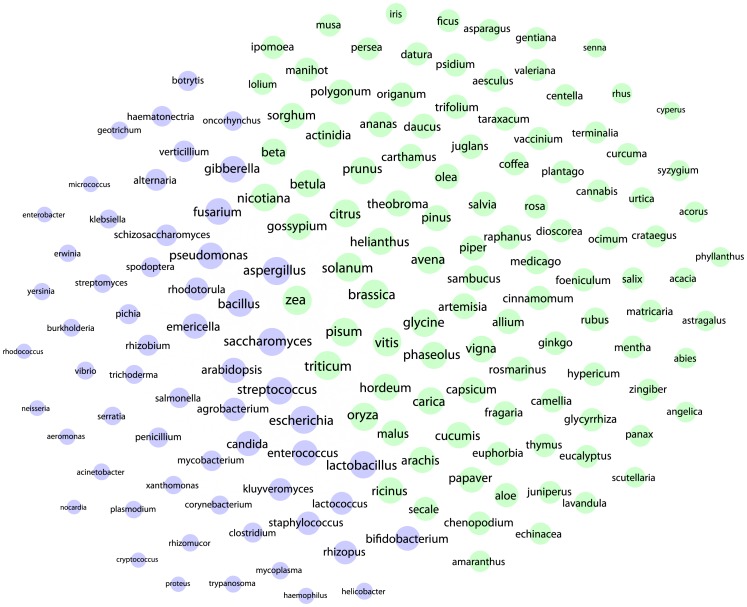
Co-occurrence Network of Top Genera in Patent Claims. This figure displays a Fruchterman-Reingold representation of the co-occurrence linkages between top genera appearing in patent claims with node size based on degree. Green indicates genera falling within Plantae. Network visualization performed in Gephi.

**Figure 3:**
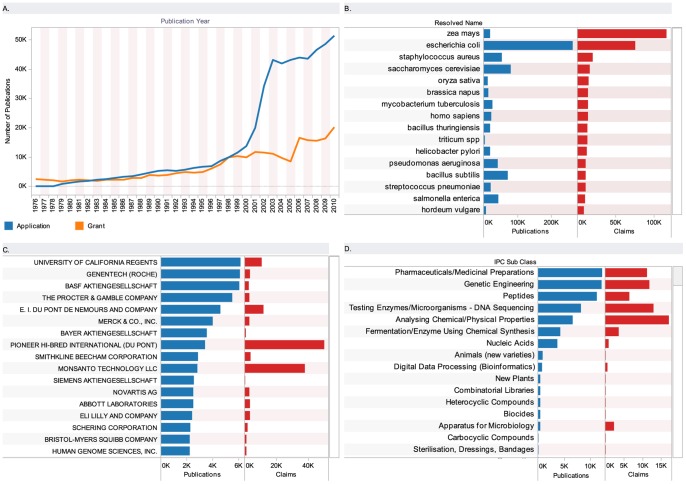
Trends in Patent Activity for Biodiversity. A. Trends by patent applications and grants based on patent kind codes A (applications) and B (grants) adjusted for US practice. Note that prior to 2001 the United States only published patent documents when granted. This produces a reporting effect in the form of a sharp spike in patent applications in 2001. B. Species appearing in patents including the number of publications (publications) where a species appears and the number of occurrences of a species name in the patent claims (claims). The data is ranked on occurrences of a species in patent claims as a measure of the intensity of activity for the species. Note that data for food crops includes common names (Supporting information S1). C. Patent applicants harmonized using EEE-PPAT 2012 (Supporting information S1). Data does not address mergers and acquisitions. D. Technology area based on International Patent Classification, 8th edition, sub-class codes. Sub-class descriptions have been edited for presentation.

In total we identified 767,955 patent documents originating from 354,003 patent families (first filings) that contain references to species (Workbook S1, table S3). We identified 25,495 species names in the claims section of 136,880 patent documents (Workbook S1, table S3.1, S3.2). Figure 2 is a co-occurrence network map of the top genera appearing in patent claims and provides a basic guide to the dominant genera discussed in this article. Figure 3 summarizes overall trends in activity between 1976 and 2010 (Supporting information S1). We approach key features of the landscape by the major technology areas identified in Figure 3D.

### Pharmaceuticals and Medicines

The role of patent protection in the development of pharmaceuticals and medicines is an important subject of debate because of its impacts on the cost of medicines for consumers and health care systems and the orientation of medical research. It is important to emphasize that our data focuses on organisms associated with particular conditions rather than specific conditions i.e. cancers. For ease of explanation we organize the results into categories of conditions rather than the rank of individual organisms in the patent data (Workbook S1, table S4).

The top species that are a target of activity are almost exclusively bacteria led by organisms associated with hospital infections, infections in medical devices and in individuals with weakened immune systems. The top target species are *Staphylococcus aureus*, for Methicillin Resistant Staphylococcus Aureus (MRSA) hospital infections, *Pseudomonas aeruginosa*, *Candida albicans*, *Clostridium dificile* and *Enterococcus faecalis*. The prominence of antibiotic resistant bacteria suggests that innovations involving biodiversity in the patent system are frequently directed to controlling problems created by earlier biotechnological innovations.

A second category of disease agents are familiar as respiratory infections, notably: *Mycobacterium tuberculosis* (tuberculosis), *Streptococcus pneumoniae* (pneumonia), *Haemophilus influenzae* (pneumonia), *Streptococcus pyogenes* (scarlet fever), *Chlamydophila pneumoniae* (pneumonia), *Bordetella pertussis* (whooping cough), *Mycobacterium avium* (for late stage AIDS patients), *Mycobacterium bovis* (for cross-species TB infections) and *Klebsiella pneumoniae* (pneumonia) (Workbook S1, table S4).

A mixed third category includes *Helicobacter pylori* (gastritis/ulcers), *Salmonella enterica* (food poisoning), *Neisseria meningitidis* & *Enterococcus faecium* (meningitis), *Chlamydia trachomatis* (trachoma or blindness), *Borrelia burgdorferi* (Lyme disease), *Neisseria gonorrhoeae* (Gonorrhoea), species of Human papillomavirus (cervical cancer) and *Porphyromonas gingivalis* (periodontitis) (Workbook S1, table S4).

Patent activity for species associated with bioweapons/bioterrorism is typically targeted at controlling or treating pathogens including: *Bacillus anthracis* (anthrax), *Clostridium botulinum* (botulism), *Yersinia pesti*s (plague), *Variola major* (small pox), *Francisella tularensis* (tularaemia) and Ebola virus (i.e. *Zaire ebolavirus*) for viral haemorrhagic fevers (Workbook S1, table S4).

Neglected tropical diseases are an increasing focus of international attention because of the burdens imposed by these diseases on the economies and welfare of the populations of developing countries [100]. These diseases are also of increasing relevance to developed countries because of the increasing mobility of human populations and the potential impacts of climate change [101], [102]. It is frequently assumed that neglected tropical diseases are of limited interest to companies and research organisations involved in patent activity because of the economic status of the victims of these diseases and the ability of national health systems to pay for treatment. Our research reveals a more complicated picture of neglected tropical diseases in the patent system.

We identified 95 species from 64 genera associated with five categories of neglected tropical diseases in 67,536 patent documents (Workbook S1, table S5). The data is dominated by bacterial infections that are of shared concern in developed and developing countries such as *M. tuberculosis*, *M. bovis*, *S. enterica* and *Vibrio cholerae* (cholera). For protozoa activity is focused on *Leishmania* species (leishmaniasis) and the trypanosomes (i.e. *Trypanosoma cruzi* for Chagas disease and *Trypanosoma brucei* for sleeping sickness) [103], [104]. Activity for helminth (parasitic worm) infections involves 31 genera and is concentrated on schistosomas (notably *Schistosoma mansoni*), *Taenia solium* (taenia), *Fasciola hepatica* (liver fluke) and *Onchocerca volvulus* (onchocerciasis or river blindness) [105]. Patent activity for Mycetoma (fungal infections) is led by *Acremonium strictum* and *Paracoccidioides brasiliensis* followed by *Pseudallescheria boydii* [106]. The remaining 7 species in the Mycetoma group display low activity (<195 publications per species). Activity for ectoparasitic organisms is dominated by a Botfly *Oestrus sp.* (i.e. *Oestrus ovis*, a fly larvae infection in sheep) and *Sarcoptes scabiei* (scabies). The remaining Myiasis related Botflies each display <800 publications per species [107].

The data reveals that patent activity for neglected tropical diseases has increased significantly and is strongly associated with the rise of biotechnology. However, we would caution that when shared areas of concern between developed and developing countries, such as *M. tuberculosis* and *C. trachomatis*, are deducted total activity for neglected diseases is lower than for *S. aureus* as a single species (Workbook S1, table S5.1). Nevertheless, patent data presents an important potential avenue for identifying effective treatments for neglected tropical diseases. Initiatives to advance research in this area include a patent pool known as WIPO *Re:search*. WIPO *Re:search* was established by the World Intellectual Property Organization to promote the sharing of intellectual property relevant to neglected diseases. The World Intellectual Property Organization has also commissioned a series of patent landscapes on issues of relevance to developing countries under the WIPO Development Agenda. In our view, countries such as Brazil, India and China with major scientific research capacity could also consider doing more to deploy this capacity to maximize positive impacts upon the welfare of their populations and those of neighbouring countries [108]–[113].

So far we have focused on organisms that are associated with particular medical conditions. However, natural products play a key role in the development of approved pharmaceuticals [114]. It has widely been reported that natural products are a declining focus of interest for R&D investments by major pharmaceutical companies with many companies moving out of this sector in the 1990s [74], [115]. However, a more complex picture emerges in the patent data. We focus on here on plants in organic pharmaceutical preparations.

We identified 10,080 plant names from 2,813 genera in 31,621 publications in the collections searched (Workbook S1, table S6). The top ranking species are: *Aloe vera* for dermatological disorders and antineoplastic agents; *Ginkgo biloba* for nervous system disorders; *Taxus brevifolia* (Pacific yew) for antineoplastic agents; *Cannabis sativa* (cannabis) for nervous system disorders; *Camellia sinensis* (tea) for antineoplastic agents and anti-infectives; *Panax ginseng* for a range of agents including antineoplastic agents; *Vitis vinifera* (grape vine) for dermatological disorders; *Momordica charantia* (Bitter melon) for antineoplastic agents and hyperglycaemia; *Curcuma longa* (turmeric) for antineoplastic and dermatological disorders; *Glycyrrhiza glabra* (Liquorice) for dermatological disorders; *Glycine max* (soybean) for urinary system disorders; *Centella asiatica* (Indian pennywort) for dermatological and anorexiant/anti-obesity agents; *Hypericum perforatum* (St. Johns wort) for antidepressants and anxiolytics; *Camptotheca acuminata* (the Chinese happy tree or cancer tree) for antineoplastic, antiviral and anti-parasitic agents; *Zea mays* (maize), in connection with anti-infectives, and *Rosmarinus officinalis* (rosemary) for dermatological disorders.

The presence of plants in pharmaceutical preparations is strongly associated with traditional medicines as a growing area of patent activity [116], [117]. We identified 12,045 plant species and 1,519 species of fungi in activity for traditional medicines (Workbook S1, table S7). Activity is dominated by the widely known plant species highlighted above. Other top plant genera include *Turnera* (Passifloraceae), *Pinus* (pines), *Solidago* (goldenrods), *Opuntia* (cactus) and *Viola* (violet family). An estimated 513 genera of fungi appear in traditional medicine patents (Workbook S1, table S7). Looking beyond fungi associated with antibiotics, pathogens or biotechnology, fungi are dominated by common *Ganoderma*, *Agaricus* and *Trichophyton* species. Less familiar species include *Grifola frondosa* and endoparasitic *Cordyceps sinensis* and *Cordyceps militaris* to address a range of conditions including diabetes, anorexia/obesity, cancers and transplant rejection.

Cosmetics is a strong cross-over area with pharmaceutical and medicinal preparations. The top genera are dominated by *Aloe*, *Camellia* (tea), *Citrus*, *Prunus* and *Mentha* but extend to unusual species of *Coffea* from countries such as Madagascar (Workbook S1, table S8, S9) [118]. The top patent applicants for cosmetics involving claims relating to plants include Proctor & Gamble, L’Oreal, Henkel, Unilever, Beirsdorf, Shiseido and Kao Corp (Workbook S1, table S10). This raises questions about the constructive contribution that these companies might make to the conservation and sustainable use of biodiversity.

Patent activity for pharmaceuticals, traditional medicines and cosmetics is associated with debates on the misappropriation (biopiracy) of genetic resources and traditional knowledge. Biopiracy can generally be understood as the collection of biological materials and recording the knowledge of indigenous peoples and local communities (traditional knowledge) that subsequently become the focus of a patent application directed to creating a commercial product. Biopiracy is a politically contested concept [77], [119]. However, its key features include a lack of prior informed consent from those providing the biological materials and knowledge of its uses and an absence of benefit sharing. In international policy debates biopiracy links issues of state sovereignty over biological resources with the human rights of indigenous peoples and local communities [77], [120]–[122]. The 2010 Nagoya Protocol is intended to address the problem of biopiracy through a requirement for prior informed consent from countries and indigenous and local communities where collections of material and associated traditional knowledge take place. As part of this process the Nagoya Protocol also requires the establishment of mutually agreed terms on benefit-sharing in access and benefit-sharing contracts as a precondition for access to genetic resources and traditional knowledge for research and development [78], [79], [123]. Special provision is made in Article 8(a) of the Nagoya Protocol to promote non-commercial research while taking into account the need to address change of intent towards commercial research.

As the Nagoya Protocol moves towards ratification companies and researchers will need to adjust to these new requirements [79], [124]. This is likely to include a requirement for benefit-sharing arising from the pursuit of intellectual property and checks on compliance with the terms of access and benefit-sharing contracts and permits. The overall aim of the Nagoya Protocol is to generate benefits for the conservation and sustainable use of biodiversity. However, this raises the question of what contributions companies and researchers seeking to commercialise biodiversity and traditional knowledge might make to the conservation and sustainable use of biodiversity and enhancing the welfare of indigenous and local communities. In short, access to biodiversity and traditional knowledge is not free but increasingly carries with it responsibilities and obligations.

**Figure 4:**
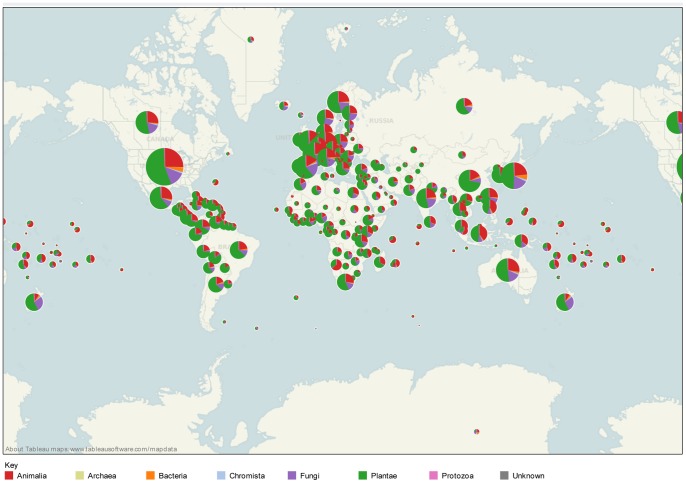
Global Distribution of Species in Patents by Kingdom. This figure is based on available distribution data from GBIF. Note that GBIF data may be incomplete.

The Nagoya Protocol is predicated on the idea that individual countries possess unique biodiversity and traditional knowledge that may prove to be commercially valuable. That is, that a particular species is in a sense ‘owned’ by a country while traditional knowledge is special to the indigenous peoples and communities concerned. In some cases species may be unique to a particular country. However, species do not respect political boundaries and are frequently distributed in more than one country. Figure 4 displays the distribution of species appearing in patents by kingdom based on available distribution data from GBIF. As the lists of species presented above reveal, the bulk of patent activity is concentrated around a small number of well-known and cosmopolitan species. This appears to reflect a ‘herd like’ tendency among patent applicants. Species that are limited to one or a very small numbers of countries are likely, on the basis of available distribution data, to be exceptions rather than the rule. It is important to balance this observation with recognition that local adaptations to environmental conditions may result in a particular sample possessing distinct properties when compared with other members of the species or genus elsewhere in the world. Examples in this area would include distinct strains of *Streptomyces* species for use in antibiotics or distinct plant varieties such as *Oryza sativa Gigante* for use in conferring resistance to rice yellow mottle virus in agriculture.

Species from biodiversity rich developing countries are an increasing source of the diversification of species within the patent system. Our data suggests that these species generally appear at lower frequencies than top ranking and widely distributed species. Examples within the data include *Boswellia serrata* (India), *Camptotheca acuminata* (China and Tibet), *Carpotroche brasiliensis* (Brazil) and *Sclerochiton ilicifolius* (South Africa). In some cases, such as *Camptotheca acuminata* (the source of the semi-synthetic topotecan anti-cancer treatment *Hycamtin*) a species may be the source of a blockbuster drug [125]. In others, such as *Sclerochiton ilicifolius* as the source of the super sweetener Monatin, the economic potential of a natural product may become apparent in the near future [126], [127]. What remains to be investigated is the extent to which species originating from these countries become the basis for commercially valuable products.

The emergence of traditional medicines and other natural products in the patent system that originate from biodiversity rich developing countries is one source of diversification in the patent system. Typically these take the form of raw extracts or partially or wholly characterized compounds. However, a second source of diversification is provided by genetic engineering, biotechnology and genomics to which we now turn.

### Genetic Engineering

Genetic engineering is a major focus of activity across a spectrum of sectors. The top results by species provided in Figure 3B are closely associated with the rise of biotechnology and genomics (Workbook S1, table S11). This is revealed through the prominence of species such as *Zea mays* (maize), *Escherichia coli*, *Saccharomyces cerevisiae*, *Oryza sativa* (rice), *Bacillus thuringiensis* and *Bacillus subtilis*. These organisms are frequently model organisms that are used to explore the fundamental genetics of a range of organisms and have become important research tools for use in biotechnology (e.g. *E. coli*). They are accompanied by model organisms such as *Homo sapiens*, *Caenorhabditis elegans* (nematode worm), *Drosophila melanogaster* (fruit fly) and *Arabidopsis thaliana* (Thale cress).

Biotechnology is a key driver of diversification in the use of genetic resources. In contrast with traditional medicines, biotechnology involves diversification in the patent system at different levels of the evolutionary tree. For example, towards the base of the tree Hox genes are involved in the budding of limbs and other morphological features in embryos [128]. In contrast, on the outer branches of the tree Single Nucleotide Polymorphisms (SNPs) may confer resistance to disease [129]. Patent activity in biotechnology, including emerging fields such as synthetic biology, is narrowly focused around a small cluster of species that provide insights into the fundamental genetics of wider groups of organisms. At the same time, this focus on the fundamental genetics of organisms may permit the extension of patent claims across multiple organisms based on their shared evolutionary history [130]. Examples in this area include primate embryonic stem cells and the rice genome [54], [130].

Patent activity involving the human genome is a key focus of controversy in connection with ethics and the implications of patent activity for research involving humans [27], [131]–[133]. Consistent with our focus on Latin names we searched for members of *Homo* in patent data. We identified 11,204 original filings linked to 22,418 patent publications in the major collections focusing on members of *Homo* (Workbook S1, table S12). References to *Homo sapiens* are heavily focused on DNA sequencing, peptides and potential treatments for cancer (Workbook S1, table S13). Activity is led by Bayer Healthcare, Incyte Genomics, Isis Pharma, the University of California and Genentech (Hoffmann-La Roche) (Workbook S1, table S14). Our data captures well-known controversies involving patents for primate embryonic stem cells involving the Wisconsin Alumni Research Foundation (WARF), breast cancer involving Myriad Genetics and erythropoietin involving Amgen for the treatment of anaemia [134]–[136].

One increasingly prominent feature of debates on DNA patents is the question of whether DNA, as a product of nature, is patentable subject matter [137]. This question is distinct from the question of whether the isolation of a DNA sequence from its natural environment and identification of its properties constitutes an inventive step [137]. In November 2012 the United States Supreme Court decided that it would consider the question “Are human genes patentable?” under Article $101 of US patent law (USC 35) in the long running case *Assoc. for Molecular Pathology v. Myriad Genetics, Inc.* involving human DNA sequences used in diagnostic testing for susceptibility to breast cancer [138]. In June 2013 the Supreme Court decided that “genes and the information they encode are not patent eligible under $101 simply because they have been isolated from the surrounding genetic material” [139]. However, the Court also decided that synthetic DNA (cDNA) is patentable subject matter. This decision has reportedly resulted in confusion for patent holders [140]. It is unclear whether other countries outside the United States will take a similar view and restrict patent claims over isolated DNA except where the DNA has been modified.

The genetic engineering of foods is a second major area of debate. The majority of the world’s population depend on just ten staple food crops for their food security: maize, rice, wheat, potato, soybeans, cassava, sweet potato, sorghum, yams and plantain [141]. The positive and negative impacts of genetically engineered foods (GM foods) are heavily contested and opinion differs sharply on whether GM foods can contribute to enhancing food security in developing countries or whether dependence on technology inputs and their associated costs undermines food security and livelihood systems [142]–[144]. Opinions also diverge on whether GM crops undermine the natural genetic diversity essential for the long-term capacity for agricultural innovation in the face of change [145].

The top species for genetic engineering and agriculture are *Zea mays* (maize), *Oryza sativa* (rice), *Brassica napus* (oilseed rape/canola, used as oil and fuel), *Triticum sp.* (wheat), *Hordeum vulgare* (barley), *Helianthus annuus* (sunflower for use as oil) followed by *Solanum tuberosum* (potato), *Brassica oleracea* (the ancestor of cabbage, cauliflower, broccoli and Brussels sprouts), *Sorghum sp.* (sorghum) and *Pisum sativum* (pea) (Workbook S1, table S15). Patent activity for food crops is concentrated in a small number of companies led by Du Pont (including Pioneer Hi-Bred), Monsanto, Bayer, BASF and Syngenta (Workbook S1, table 16) [146], [147]. This raises the question of whether companies are focusing on genetic engineering at the expense of more promising avenues of enquiry to address food security. The narrowness of agricultural R&D could also lead to a failure to appreciate the strategic importance of *in situ* agricultural biodiversity and *ex situ* collections under the International Treaty on Plant Genetic Resources for Food and Agriculture [69], [148].

The concentration of agricultural R&D in a small number of major food crops raises questions about the role of neglected and underutilized species (NUS) or ‘neglected foods’ in addressing food security needs [149], [150]. The *Crops for the Future* initiative lists 893 plant species as neglected and underutilized (Workbook S1). 527 (59%) of these species appear in 39,900 patent publications originating from 18,374 original filings in our data (Workbook S1, table S17). At first sight it would appear that the majority of these species are not neglected in the patent system. However, patent activity is heavily concentrated in species such as *Aloe vera* and *Morinda citrifolia* and directed towards medicines and cosmetics rather than food (Workbook S1, table S18).

Our research suggests that a longer term strategic approach to enhancing food security would focus on promoting the value of the world’s plant genetic resources both *in situ* and in *ex situ* collections and diversification of the species involved in R&D to focus on a wider range of species including neglected and under utilized species.

The International Treaty on Plant Genetic Resources for Food and Agriculture has established a multilateral access and benefit-sharing system for access to the world’s major *ex situ* collections of plant genetic resources for major food crops and forages. The multilateral system is organized around a Standard Material Transfer Agreement (SMTA) setting out the terms of benefit-sharing arising from the use of plant genetic resources under the system. Under this system countries make their collections of plant genetic resources available for wider use on the condition that benefits arising from commercial utilization are shared through a global fund for the purposes of conserving plant genetic resources. To date this has taken the form of small grants to farmers in developing countries directed to conserving agricultural biodiversity. In 2008 the first community organization, the Potato Park in the Peruvian Andes established by six Quechua communities, became a member of this system to conserve the genetic diversity of the potato. The creation of this nascent global benefit-sharing mechanism is also inspiring wider debates on the possibilities of creating a global commons for crop genetic resources that both reflects and supports the traditional exchange of agricultural resources among farmers around the world and supports the objectives of the International Treaty [52]. However, the SMTA system is relatively new and will take time to generate benefits due to the length of time involved in plant breeding. For this reason the benefit-sharing fund established under the Treaty is presently funded by voluntary contributions from member states rather than users of the multilateral system [151]. This suggests that further work and innovative approaches will be required to secure funding for the conservation of the world’s plant genetic resources. Part of the difficulty with securing investments from industry and other users of plant genetic resources is the difficulty of tracing the use of accessions from the multilateral system both inside and outside the patent system [151]. Further work in this area could contribute to firmly establishing the principle and practice that users must pay. This could perhaps be linked with bolstering incentives for local conservation through payments for ecosystem services (PES) [152].

### Biocides

Killing biodiversity is an important feature of patent activity. Approximately 29,129 species appear in 57,227 patent documents for biocides and can be divided into research tools and target species (Workbook S1, table S19). Top research tools in this area include *E. coli* and *B. thuringiensis*. Important target species include *S. aureus*, *Botrytis cinerea* (a neotropic fungus), *Blumeria graminis* (a fungus affecting cereals), *Phytophthora infestans* (potato blight), *P. aeruginosa*, *Magnaporthe grisea* (rice blast fungus) and *Plasmopara viticola* (mildew in grapevines). Top animal species include *Musca domestica* (common housefly), *Tetranychus urticae* (plant feeding spider mites), *Leptinotarsa decemlineata* (Colorado potato beetle), *Nilaparvata lugens* (brown planthopper rice pest), *Blatella germanica* (German cockroach) and *Plutella xylostella* (diamond back/cabbage moth).

The top patent applicants for biocides include Bayer, BASF, Ciba Geigy, Syngenta, Du Pont and Sumitomo Chemicals (Workbook S1, table S20). This area of technology is dominated by heterocyclic and carbocyclic compounds and includes the use of *Streptomyces* and *Penicillium* (as sources of antibiotics). Genetic engineering and peptides represent significant growth areas in recent years led by Mycogen, Syngenta, the University of California and Novartis. There is a strong association between companies in the agricultural sector and biocides but this sector also includes pharmaceutical companies and major chemical companies. A key issue arising from patent activity for biocides is the wider impact of compounds, including antibiotics, on biodiversity and human health [153]–[155]. A recent example in this area is the controversy over the impact of neonicotinoids upon bee populations in Europe [156]–[158].

### Marine Organisms & Extremophiles

Marine genetic resources are an important focus of R&D across a range of marine environments [159]. Using the Ocean Biogeographic Information System (OBIS) database we identified 4,162 marine species in patent data of which 1,464 species appear in patent claims (Workbook S1, table S21). Important marine species include the familiar *S. cerevisiae* and *V. cholerae* followed by the bioluminescent sea pansy (*Renilla reniformis*) and the jellyfish *Aequorea victoria* as sources of Green Fluorescent Protein (GFP) for use in biotechnology [160], [161]. Other important species include *Dunaliella salina* (a green micro algae) for use in cosmetics and dietary supplements [162]. *Crypthecodinium cohnii* is a red microalgae that is a source of Docosahexaenoic acid (DHA) widely used as a food supplement in infant formulas [163]. *Serenoa repens* (saw palmetto) is a coastal palm that was historically used by Native Americans and more recently in managing prostatic hyperplasia [164]. *Perna canaliculus* is the New Zealand green lipid mussel used in research on a lipid extract in patients with prostate and breast cancer and in complementary therapy for osteoarthritis [165], [166]. *Limulus polyphemus* (Atlantic horseshoe crab) is associated with research on vision that led to the 1967 award of the Nobel Prize for Medicine [167]. *Chondrus crispus* is a red algae found along shorelines in Northern Europe that is a source of carrageenan used as a thickener in milk products [168].

Research has increasingly penetrated to the deep sea bed beyond national boundaries governed under the United Nations Convention on the Law of the Sea (UNCLOS) [88], [169]. Using the *Chemosynthetic Ecosystem Science* (ChEssBase) database of 1,085 marine organisms with additional research we identified an initial 128 deep sea and hydrothermal species in the patent data (Workbook S1, table S22). Patent activity is led by extremophiles such as *Thermus thermophilus*, *Methanocaldococcus jannaschii*, *Thermococcus litoralis*, *Aeropyrum pernix*, *Archaeoglobus fulgidus* and *Pyrococcus horikoshii*. Other species include the giant tube worm *Riftia pachyptila* for a novel fusion protein and *Beryx splendens* (the Splendid alfonsino fish) for foodstuffs and medicines. *Anoplopoma fimbria* is involved in the development of vaccines for fish and *Bythograea thermydron* (the vent crab) for a new nucleic acid useful in therapeutic insertions of DNA. *Microstomus pacificus* (Pacific dover sole) is involved in a claimed new treatment for bone disorders.

Research in Antarctica is governed under the Antarctic Treaty [90]. We identified 439 species in patents that are recorded by GBIF as occurring in Antarctica (Workbook S1, table S23). For example, *Pseudozyma antarctica* is a component in the production of Itaconic acid for use in paper and coatings. *Dissostichus mawsoni* (Antarctic cod) has been a focus of interest for anti-freeze proteins, addressing tissue destruction in cryosurgery, and cosmetics. *Deschampsia antarctica* (an Antarctic grass) has fuelled activity for an ice recrystallization inhibition protein, a biofertiliser, and an extract with activity for colon cancer. *Synoicum adareanum* and *Aplidium cyaneum* are a focus of activity in potential cancer treatments while *Chaenocephalus aceratus* (white blooded icefish) is a focus of activity for hematopoietic genes for the diagnosis and treatment of sickle cell anaemia. Finally, Airbus references *Pagothenia borchgrevinki* as a source of anti-icing proteins for wings, rotors and turbines (Supporting information S1, table D).

These examples illustrate the diversity of potential applications arising from marine organisms and extremophiles but raise concerns about benefit-sharing and the environmental impacts of research in these environments [90], [169], [170]. We would emphasize that further research is required to provide a more comprehensive picture of patent activity involving organisms from these environments and the impacts of research.

## Discussion

Biological diversity is fundamental to the human capacity to innovate to address existing and future challenges. However, patent activity is focused on a very narrow segment of taxonomically described biodiversity and approximately 1% of anticipated biodiversity on this planet. This narrow concentration of R&D activity and ownership is unlikely to be in the long terms interests of humanity.

Nature will rarely be beaten as a source of new and useful products. Yet, we know relatively little about the diversity of life on this planet. Our research suggests that a new strategic approach is required to R&D investments involving biodiversity. We now consider three basic interlocking elements for a strategic approach: 1) taxonomy; 2) research and development, and; 3) intellectual property and incentives. In the process we discuss key aspects of each element where action is required by different actors.

### Taxonomic Research

The first element in a strategic approach is that taxonomic research, broadly conceived, and biodiversity informatics should receive greater priority and investment. This has two main aspects. The present research is grounded in the growing availability of taxonomic data from collections around the world under the umbrella of the Global Biodiversity Information Facility (GBIF). These contributions are complemented by the work of the Catalogue of Life, the Encyclopedia of Life and related initiatives such as the Biodiversity Heritage Library. Greater investment is required in these initiatives to enhance the availability of digital taxonomic and biodiversity information to advance our understanding of biodiversity and its role in human innovation. In our view, countries around the world should promote the inclusion of taxonomic data in GBIF as part of an effort to address the problem of scale in access to basic information about biodiversity on Earth.

The second aspect involves tackling the poverty of scientific knowledge of biodiversity. The ability to know and understand biodiversity is fundamentally dependent on the availability of people on the ground who are willing to record biodiversity and assess habitats and ecosystems. This is not a matter of simply increasing the numbers of professional biologists and taxonomists. Mobilizing and securing public participation is vital. This involves a willingness to reach across professional boundaries, languages and cultural and spiritual values. This is particularly important in the case of indigenous peoples and local communities who frequently inhabit the most biodiverse and threatened areas of this planet. The Global Taxonomy Initiative under the Convention on Biological Diversity increasingly recognizes that indigenous peoples and local communities require support in conserving and making their taxonomic knowledge available on terms that recognize and are respectful of their rights (COP Decision IX/22 & X/39). Advances in recognition of the human rights of indigenous peoples such as the 2007 *United Nations Declaration on the Rights of Indigenous Peoples* and within the Nagoya Protocol represent an important step forward in addressing this problem [171]. However, it appears unlikely that indigenous peoples and local communities will be able to fully participate in advancing taxonomic knowledge in the absence of practical measures by governments to provide conditions of reasonable certainty that their rights and interests will be respected.

Promoting public participation in taxonomy and biodiversity extends to promoting wider citizen participation in both developed and developing countries. The growing availability of digital tools and the rise of ‘citizen science’ provide major avenues for mass public engagement in enhancing knowledge of biodiversity and raising wider awareness of the importance of biodiversity for humanity [172]–[174].

### Research and Development

The second element in a strategic approach is understanding and addressing concerns about biopiracy and misappropriation with the objective of promoting research and development. In biodiversity rich developing countries the spectre of biopiracy casts a long shadow over basic research on biodiversity. Concern about biopiracy is tied up with the historical experience of expropriation of natural and biological resources, such as rubber and tea, in the colonial period [72], [175], [176]. The rise of intellectual property claims over biological diversity and traditional knowledge from the 1990s onwards has given the impression that colonial practices are continuing through other means. In response, developing countries have asserted their sovereignty over biological and genetic resources. The collision between claims to state sovereignty and the internationalization of intellectual property rights by developed countries has produced a situation described as ‘hyperownership’ in the realm of biology [29]. This situation closely corresponds with an ‘anticommons’ characterized by a multiplicity of claims to property and expectations of value that stifle opportunities for sharing and innovation [27], [177]. In key respects claims to state sovereignty over genetic resources and intellectual property claims depend on the perception of potential, rather than actual, economic value. This produces a perverse situation. Biodiversity has become a battlefield over the ability to capture the potential economic values of genetic resources and traditional knowledge. At the same time, biocultural diversity is gravely threatened by human activity and the failure of governments to take adequate and effective action to conserve biodiversity and promote respect for the rights of indigenous peoples and local communities [178]–[180].

The Nagoya Protocol is intended to contribute to addressing these problems through the emphasis it places on prior informed consent and establishing mutually agreed terms on benefit-sharing with governments and affected indigenous peoples and local communities in return for access to genetic resources. Specifically, it is hoped that the Nagoya Protocol will improve levels of legal certainty over respect for the rights and interests for indigenous peoples and local communities, governments and non-commercial and commercial research involving genetic resources and traditional knowledge. Establishing a situation of reasonable certainty will however, involve adjustments to the practices and expectations of participants.

Under the Nagoya Protocol, researchers should expect to encounter more stringent conditions for collecting samples in the terms of permits, access and benefit-sharing contracts and a new “international certificate of compliance” (Nagoya Protocol Article 14.4 & 17). Conditions are also likely to be attached to the transfer of materials to third parties including provisions for change of intent from non-commercial to commercial use and requirements for pursuing intellectual property rights. The emphasis here is likely to be placed on ensuring benefit sharing, in non-monetary and monetary forms, with the original providers of genetic resources and traditional knowledge. Researchers who will be engaging with indigenous peoples and local communities, including research on their lands or territories, should expect to see a greater emphasis on research ethics, codes of conduct and what the Nagoya Protocol calls community protocols. The latter are expected to set out community requirements and expectations of research as a condition for working with indigenous peoples and local communities.

Professional researchers are accustomed to complying with rules. In the case of research involving indigenous peoples and local communities requirements for ethical conduct are a standard part of professional practice in the social sciences and biomedicine. A range of guidance and best practice exists for establishing ethical and respectful relationships with indigenous peoples and local communities [181]–[184]. Key principles in research with indigenous peoples and local communities include respect for rights, participation throughout all stages of research, agreement on intellectual property considerations and sharing in the benefits of research.

On the national level, in the early days of the Nagoya Protocol it is likely that researchers will confront rules and processes that are, for all practical purposes, impossible to navigate. This will frustrate researchers and frustrate the purposes of the Nagoya Protocol. Countries will need to recognize that practical rules will be to their long-term advantage in promoting international collaborations and investments directed to both non-commercial and commercial research involving biodiversity. Here a balance will need to be struck between seeking to protect potential future benefits, which may be unrealizable, and the actual benefits of international research collaborations in terms of funding and technology and knowledge transfers. What matters here is that research is promoted to enhance knowledge and understanding of biodiversity while recognizing and taking appropriate measures to address potential commercial research and development. In our view, expectations about the potential value of genetic resources and traditional knowledge should not be allowed to hold the actual value of international research collaborations hostage.

In so called ‘user’ countries, such as member states of the European Union, public funding agencies have an important role to play in promoting respect for the spirit and letter of the Nagoya Protocol within the scientific community [185]. Specifically, guidance is likely to be required for university recipients of public funding on meeting obligations under the Protocol. Universities are important actors in the patent landscape for biodiversity and are increasingly encouraged to pursue the commercialization of research through patents and commercial contracts. We anticipate that universities that are serious about promoting international collaborations in research involving biodiversity will need to ensure compliance with obligations to secure prior informed consent and mutually agreed terms on benefit-sharing under the Nagoya Protocol.

University obligations will also extend to future utilizations of genetic resources. Article 2(c) of the Protocol establishes that it applies to “research and development on the genetic and/or biochemical composition of genetic resources, including through the application of biotechnology…”. As such, the Protocol applies to any research and development on the genetic or biochemical properties of genetic resources, excluding human genetic resources, that occurs following the entry into force of the Protocol. Universities will therefore need to ensure that follow on utilizations of genetic resources and traditional knowledge, i.e. through transfers or licensing to third parties, respect the terms of access and benefit-sharing agreements and the Nagoya Protocol.

A significant, if presently unknown, proportion of taxonomically described biodiversity is held within public and private collections established prior to the Nagoya Protocol. In cases where the material was acquired prior to the entry into the force of the Protocol, or it is not possible to obtain prior informed consent, Article 10 of the Protocol anticipates that benefit-sharing may be channelled through a Multilateral Benefit-Sharing Mechanism. The form that this benefit-sharing mechanism might take is under discussion and may, as with the Plant Treaty (see above), create a common pool of genetic resources that can be shared with limited conditions. It will be important for both universities and companies to clarify the provenance of existing genetic resources within their collections in preparing for the entry into force of the Protocol and to consider their own possible contributions to a Multilateral Benefit-Sharing Mechanism from their own collections. A number of major public collections, including botanic gardens and museums, actively participated in the negotiation of the Nagoya Protocol and are familiar with its terms. Under proposals being considered within the European Union, public collections may in future take on the status of “trusted collection” as sources of genetic materials that comply with the Protocol [185]. The major collections are likely to specify terms in material transfer agreements that comply with the Nagoya Protocol on non-commercial and commercial use. At the same time, the major collections, such as Kew Gardens, have developed guidelines and information resources such as the *Principles on Access to Genetic Resources and Benefit-sharing for Participating Institutions* to assist collections with adapting to the Nagoya Protocol.

Companies mainly participated in the negotiation of the Nagoya Protocol through representatives of trade associations, notably the International Chamber of Commerce and PhRMA among others. Company representatives typically emphasise a willingness to comply with rules set by governments provided that they know what they are and, preferably, that they are workable from a business perspective. In practice, companies engaging in direct collection of samples will need to pay close attention to national legislation and regulations on access and benefit-sharing. In many cases we suspect that companies will typically source genetic material from third party suppliers either inside or outside the provider country. Because the Nagoya Protocol applies to follow on uses of genetic material provided by a contracting country this will mean that companies will need to review their supply chains to check compliance with national legislation on access and benefit-sharing and the Protocol. Examples of emerging best practice in promoting compliance in the industry sector already exist. One example of emerging best practice is provided by the 2010 Union for Ethical Biotrade *UEBT Principles on Patents and Biodiversity* developed to assist companies in the cosmetics sector with complying with the provisions of the Convention on Biological Diversity and the Nagoya Protocol. A growing number of companies involved in biodiversity related research and product development already place an emphasis on sustainability as part of their corporate responsibility agendas. We would expect to see greater evidence of financial investments in community level conservation in countries of origin in future under the terms of the Nagoya Protocol.

### Intellectual Property and Incentives

The third element in a strategic approach focuses directly on intellectual property and incentives. The Nagoya Protocol does not directly address the contentious issue of patents and intellectual property rights. The Protocol confines itself to including possible joint ownership of intellectual property as part of benefit-sharing and the creation of checkpoints for compliance under Article 17 that may, or may not, include intellectual property offices. Nevertheless, intellectual property was at the forefront of many negotiators minds in agreeing the Protocol. It is likely that intellectual property will feature prominently in the terms of future permits and access and benefit-sharing contracts.

The focus of debate on intellectual property has now turned to the Intergovernmental Committee on Intellectual Property and Genetic Resources, Traditional Knowledge and Folklore (IGC) at the World Intellectual Property Organization (WIPO). In addition, debates under the Council on Trade-Related Aspects of Intellectual Property Rights (TRIPS Council) at the World Trade Organization may receive renewed impetus [95].

Government delegations participating in the IGC, including the United States, are presently negotiating a draft treaty on genetic resources, traditional knowledge and folklore with a view to establishing a diplomatic conference to negotiate the final treaty. A key point of contention in this debate is whether applicants for patent rights should be required to say where they obtained genetic resources and traditional knowledge that are material to their inventions. In the patent system this is referred to as “disclosure of origin” or a “disclosure requirement”. The key questions under debate here include: a) whether there should be a disclosure requirement at all; b) what the disclosure should contain i.e. should it include evidence of prior informed consent from a country of origin, and; c) what the consequences of failure to disclose should be for patent applicants and grant holders (i.e. revocation of the patent, fines or other sanctions) [186].

For some countries these proposals are seen as running the risk of imposing unnecessary burdens on innovation. That is, accommodating biodiversity and the Nagoya Protocol threatens to change the purpose of the patent system with longer term negative consequences for innovation. However, a second, if opaque, aspect to this debate involves the wider economics of intellectual property markets. Intellectual property protection is a source of revenue through transfers between countries arising from licensing and royalty payments. Countries both buy and sell what has been called intangible or disembodied knowledge [187]. Recent estimates suggest that global receipts from intellectual property across all areas of technology stood at USD180 billion in 2009 [187], [188]. Intellectual property involving genetic resources and traditional knowledge will represent an unknown percentage of global receipts.

The implication of demands for disclosure of origin is that developing countries would receive a share of receipts when a patent involved a genetic resource or traditional knowledge originating from their country. The economics of intellectual property markets remain remarkably opaque. It is therefore unclear what the economic impact of transfers would be on wider markets and transfers within markets. The lack of clarity on the actual economic values of biodiversity and traditional knowledge related intellectual property and related products is a major problem. This lack of clarity creates a situation of expectations about the high economic value of biodiversity and traditional knowledge among ‘provider’ countries that is pitted against the fear of exposure to the economic costs of disclosure among ‘user’ countries. This leads to unconstructive shadow boxing between states. This is exacerbated by efforts on the part of some states to use debates at WIPO as a platform for securing reform of the TRIPS agreement.

Some countries have taken the unilateral stance that a requirement for greater transparency on the origin of genetic resources and traditional knowledge in patent applications would promote respect for the Convention on Biological Diversity and legal certainty in access and benefit-sharing arrangements under the Nagoya Protocol. Increasingly both developing and developed countries are taking unilateral action to introduce disclosure requirements into their national patent laws [186]. The practical implication of the adoption of unilateral disclosure requirements by countries such as Brazil, India, China, South Africa, Norway and Switzerland among others is that disclosure of origin will increasingly become routine for companies and research organisations seeking to operate in these markets. However, piecemeal approaches are unlikely to be as efficient as a clearly defined international standard.

It is important to recall that the fundamental purpose of the patent system is the disclosure of new and useful innovations that over time become freely available for wider use by the public. In our view it is reasonable to expect that patent applicants should state where they obtained the genetic resources and associated traditional knowledge that is material to an invention. This would have the additional advantage of improving long-term knowledge and understanding of the role of biodiversity and traditional knowledge in innovation. At the same time, increased transparency would contribute to creating conditions of trust in access and benefit-sharing by clarifying what is actually happening with genetic resources and traditional knowledge.

In practical terms, much could be achieved by following the model for disclosure of federal funding in patent applications developed in the United States under the 1980 Bayh-Dole Act [186]. The Bayh-Dole system requires recipients of federal funding to include a “Statement Regarding Federally Sponsored Research and Development” setting out the source of federal funding and contract number at the beginning of a patent application. This model for disclosure has caused no reported difficulties in the patent system and could be readily adapted to improve certainty for countries that access and benefit-sharing laws are being recognized and respected. We propose that this model could be adapted as a Statement or Declaration on Access and Benefit-Sharing in patent applications involving genetic resources and associated traditional knowledge that are material to the claimed invention. Such a statement or declaration would include a reference to contracts, permits and an international certificate of compliance to clarify the terms and conditions under which materials and any associated traditional knowledge were obtained.

In addition, the 1980 *Budapest Treaty on the International Recognition of the Deposit of Microorganisms for the Purposes of Patent Procedure* requires patent applicants to deposit a sample of a microorganism with an International Depositary Authority (such as the American Type Culture Collection). Patent applicants routinely make such deposits and provide information on the accession number and origin of organisms as part of a patent application. A Budapest Treaty style model could also contribute to addressing the concerns of developing countries and improve the clarity of disclosure of the origin of genetic resources and associated traditional knowledge in patent applications.

Proposals for potential sanctions for non-disclosure cover a spectrum from halting the processing of patent applications, to revocation of granted patents or sanctions outside the patent system such as fines. However, a more flexible approach to sanctions might focus on using existing levers in the patent system, such as patent application and renewal fee schedules or tax incentives, to create Adjustable Incentive Measures (AIMS) that reward certain types of behaviour while discouraging behaviour associated with misappropriation or biopiracy [189]. In short, there may be flexibilities in the patent system that can be used to promote compliance with the Nagoya Protocol and contribute to opening up biodiversity to research and development.

Debates at the World Intellectual Property Organization are tied to perceptions of the economic interests of member states. It remains to be seen whether those countries with an important economic stake in the patent system will be willing to accept reforms or adjustments to the functioning of the system for genetic resources and traditional knowledge. However, if they do not, it appears likely that developing countries, and some developed countries, will take unilateral action to improve transparency and certainty on the utilization of genetic resources and traditional knowledge in the patent system. These measures are likely to be less effective in the short to medium term than collective action and will raise questions about the overall ability of the patent system to respond to the wider interests of member states and the broader interests of society in biodiversity and traditional knowledge.

Patents are only one form of incentive measure [190] **.** In considering the need to open up biodiversity for innovative activity there is a need to consider expanding the range of incentives available to promote R&D on biodiversity. The majority of innovations in societies around the world do not involve patent protection. Furthermore, there is an increasing trend towards networked and open innovation across a spectrum of fields, including biology, that are an increasing focus of economic analysis [49], [191]–[194]. This raises the question of how such innovations might be made more widely visible to the intellectual property system. One answer to this question is to explore and test commons and open source approaches [46], [48], [50]. Commons and open source models are best known in the fields of software, literature, and images. These models rely on copyright law and licensing to open up materials for wider use under conditions determined by the provider. Commons models also rely on a simple set menu of options (i.e. attribution, non-commercial) that address issues of scale in both time and space. The application of these types of models in the case of biology typically takes the form of ‘open access’, i.e. in the case of genome sequence data. The potential application of commons models to biological materials and traditional knowledge merits further exploration in terms of providing a greater range of options to providers of genetic resources on the terms and conditions under which material and knowledge is made available. The key issue here is providing models that encourage others to share genetic resources and knowledge in conditions of reasonable certainty that those conditions will be respected. Viewed in international perspective, that certainty can best be provided by states. Proposals for commons approaches in the realm of biology cut across a spectrum from food and agriculture, to neglected diseases, the Nagoya Protocol and emerging fields such as synthetic biology [52], [195]–[198]. Much remains to be discussed on how effective models might be applied in the realm of biodiversity and the problems this might involve. Fundamentally, what is required are models of R&D that conserve biodiversity, promote equitable benefit-sharing, and open up biodiversity for research that addresses actual and neglected areas of human need.

## Conclusion

This article has presented the results of research on the presence of biological diversity in the international patent system. This research exploited the increasing availability of electronic patent and taxonomic data to expose the major contours of the international patent landscape for biodiversity and traditional knowledge. The key objective of this research was to improve the transparency of information about biodiversity and traditional knowledge in the patent system. We anticipate that future work could focus on improving and refining methodologies. Important priorities in this area include refining data capture on synonyms and abbreviations, expanding the coverage of common names and the coverage of patent collections. As such, the present research represents a starting point for wider research to improve the transparency of biodiversity and traditional knowledge in the patent system. We make the core data arising from this research publicly available with this article to facilitate future work.

Our research has revealed that existing patent activity is narrowly focused on a small number of species. We have argued that long-term human interests will best be served by opening up biodiversity to research and development and advancing the implementation of the Convention on Biological Diversity and its Nagoya Protocol. The obstacles to achieving this goal are significant because of the global nature of the problem and the range of actors and interests involved. In an extended discussion we identified three interlocking elements focusing on taxonomy, research and development and intellectual property and incentives that are central to the development of a long term strategic approach to opening up biodiversity to research and development to serve human needs.

In 2010 the Convention on Biological Diversity adopted a strategic plan with the vision that: “By 2050, biodiversity is valued, conserved, restored and wisely used, maintaining ecosystem services, sustaining a healthy planet and delivering benefits essential for all people” (decision X/2). The Millennium Ecosystem Assessment reminds us that over the last 50 years humans have “changed ecosystems more rapidly and extensively than in any comparable period of time in human history” and that “This has resulted in a substantial and largely irreversible loss in the diversity of life on Earth” [180]. Within the context of the loss of biodiversity and climate change it is humans who are ultimately the vulnerable species [199], [200]. The human propensity for technological innovation is part of the problem that created these existential threats. It will also be part of the solution. We have argued that what is required is greater attention to opening up biodiversity to research and development to serve human needs based on the principles of equitable benefit-sharing, respect for the objectives of the Convention on Biological Diversity, human rights and ethics.

## Supporting Information

Supporting information S1 **Table D: Patents Referenced by Subject Area and Species Name.**
(DOCX)

Workbook S1 Table S1 Raw Latin Species Names. Table S2 Resolved Latin Species Names Including Unresolved Abbreviations. Table S2.1 Resolved Latin Species Names and Genera. Table S3 Patent Publication Numbers. Table S3.1 Species in Claims. Table S3.2 Publication Numbers for Species in Claims. Table S4 Technology Area: Pharmaceuticals. Table S5 Neglected Diseases. Table S5.1 Neglected Diseases comparison with Staphylococcus aureus. Table S6 Technology Area: Pharmaceuticals - Plantae. Table S7 Technology Area: Traditional Medicines, Plantae & Fungi. Table S8 Technology Area: Cosmetics - Plantae. Table S9 Madagascar: Coffea. Table S10 Cosmetics: Applicants. Table S11 Technology Area: Genetic Engineering. Table S12 Homo sapiens: First Family Filings and Publications. Table S13 Homo sapiens by Technology Area. Table S14 Homo sapiens: Applicants. Table S15 Major Food Crops and Forages. Table S16 Genetic Engineering: Applicants. Table S17 Neglected Foods. Table S18 Neglected Foods by Technology Area. Table S19 Technology Area: Biocides. Table S20 Biocides: Applicants. Table S21 Marine Genetic Resources. Table S22 Deep Sea Organisms. Table S23 Antarctica.
(XLSX)
