# Supplementary material for: Biological Diversity in the Patent System
Source: PLoS One. 2013 Nov 12;8(11):e78737. doi: 10.1371/journal.pone.0078737 (PMC3827099; doi:10.1371/journal.pone.0078737)
Supplement: Supporting information S1 — Table D: Patents Referenced by Subject Area and Species Name. (DOCX) [file pone.0078737.s001.docx]

**S1. Supporting Information**

**Methods:**

The research proceeded in six main phases.

**Collection Phase**. The full text patent collection of the United States Patent and Trademark Office from 1976 to 2010 was downloaded from Google United States Patent and Trademark Office Bulk Download (<http://www.google.com/googlebooks/uspto.html>). The US collection is divided into 2 segments: a) 1976-2010 patent grants, and: b) 2001-2010 patent applications. Note that prior to 2001 information from the United States is only available on patent grants.

We obtained the international Patent Cooperation Treaty (PCT) collection from the World Intellectual Property Organization. We obtained the European Patent Convention collection from the European Patent Office. Details of the number of documents by collection are provided in the table below.

**Table A: Patent Collections 1976-2010**

| **Collection** | **Documents** |
| --- | --- |
| United States Patent and Trademark Office | 6,998,473 |
| European Patent Convention | 2,057,114 |
| Patent Cooperation Treaty (World Intellectual Property Organization) | 1,996,842 |
| Total | 11,052,429 |

All collections were unarchived and standardized as XML (UTF-8) text. Patent number formats were harmonized to enable federation in Phase 6 (below).

**Term Phase:** We initiated research using the Species 2000 & ITIS Catalogue of Life (2010 edition) to establish proof of concept using 1.3 million taxonomic names. To improve data coverage we subsequently obtained a list of 19 million species name entries from the Global Names Index established by the Global Biodiversity Information Facility (GBIF) and Encyclopedia of Life (EOL). The Global Names Index aims to record all known spelling variations of Latin species names and canonical names. The GNI also includes some common, non taxonomically accepted names and ‘species like’ names that were processed during the cleaning phase. We removed punctuation and associated author information because only binomial names (such as *Homo sapiens*), and where available sub-species, were required. As a result of this exercise 6,081,631 Latin species names were available in the GNI for use in searching.

**Pattern Phase**: To correctly and efficiently locate species names within the patent corpus the collection of terms were formed by algorithm into sets of patterns (regular expressions). The algorithm had the following features: 1. Identify sets of terms with high commonality. 2. Capture commonalities using atomic grouping. The patterns sets were serialized into JSON, a compact text format. The pattern sets were deserialized in the indexing phase.

**Indexing Phase:** We used the Lancaster University High End Computing facility to index the species within the patent collections using pattern sets. 400 parallel processing nodes were available to use during indexing. A batch scheduler invoked a Ruby environment on each core that loaded the pattern sets and chose the patent document to index. The overall job used the map-reduce paradigm for worker nodes producing a manifest document that mapped job identifiers to a particular set of patent documents. For example the node tasked with job 1 would index the 1st, 1001st, and 2001st document with a task array size of 1000. Our corpus of documents naturally lent themselves to this form of processing. The results were stored in individual index files per job that were reduced using software tools (find, grep and xargs) into a core index.

The resulting index consisted of rows comprising species term, publication number, application and publication dates, the section of the document the term was found in (title, abstract, description or claims), and the match location in relation to the start of the segment.

**Raw Data Structure**

**Cleaning Phase.** Cleaning was required to address noise in the resulting data particularly where terms matched common words (i.e. Axis). In addition, the species based index also contained other species within its context string because more than one species may be contained in a single document or string. The major challenge involved in the cleaning step was abbreviations.

Addressing Abbreviations: One important feature in the practical use of taxonomic nomenclature is the use of abbreviations for species names i.e. *Escherichia coli* becomes *E. coli*. This presented the challenge that some abbreviations match multiple possible species names. For example *A. bevis* has 456 potential matches. Because of potential multiple matches we did not use a simple global approach to abbreviation matches (i.e. E. coli must always be Escherichia coli). Our strategy focused on the co-occurrence of an abbreviation and full species names in the same document. At its simplest, this assumes that the occurrence of *E. coli* and *Escherichia coli* in the same document signifies that the abbreviation refers to Escherichia coli. To structure this approach we developed a certainty metric as set out in the table below ranging from 1 (certain) to 4 (least certain).

**Table B: Abbreviation Resolution**

| **Resolution** | **Certainty Metric** | **Abbr. Per Publication Resolutions**  **(US,WO,EP)**  **Out of 1,426,091** | **Percentage**  **Resolution** |
| --- | --- | --- | --- |
| Full term and abbreviated form co-occur in current patent document. | 1 | 399,447 | 28.00 |
| Full term and abbreviated form co-occur in other patent documents (most frequent). | 2 | 612,508 | 42.95 |
| Single full term match in GNI term list. | 3 | 264,790 | 18.58 |
| Match from full terms in generated frequency list created by ifreq (most frequent). | 4 | 93,902 | 6.58 |
| No resolution found. | N/A | 55,444 | 3.89 |

This method resolved 96.11% of all occurrences of abbreviations. In an additional step the abbreviated names were resolved onto their corresponding full Latin species names. 6,999 unresolved abbreviations are included in the final index pending further improvements to the resolution method. We would note that further work is desirable on abbreviation matches in later steps in the method to remove extraneous noise in the form of false positives. This is an area for future refinement of the method.

Manual Review and Validation. Manual review was performed throughout the research phases to test and refine computational approaches. Review was performed using Vantage Point (64 bit) software from Search Technology Inc. Manual cleaning consisted of reviewing lists of results to identify any unresolved abbreviations retained in the index, variations in species names, and the appearance of common name artefacts. We then sought to devise computational approaches to resolve these issues. In addition, data was separately tested using samples of searches from the commercial patent database Thomson Innovation. Patent texts from Thomson Innovation were manually reviewed in Word Smith corpus linguistics software.

**Federation Phase:** The federation phase consisted of federating the results with additional offline and online databases. Federation focused on two categories of data: a) Patent data, and; b) taxonomic data sources.

**a) Patent Data**

In order to produce statistics we connected the index with the offline European Patent Office *World Patent Statistical Database* (PATSTAT, October 2011 edition). PATSTAT is the gold standard for international patent statistics. Federation with PATSTAT provided access to data including filing country, publication country, patent years, INPADOC patent families, applicants (assignees) and information on technology areas through International Patent Classification codes.

Patent Number Harmonization: Patent numbers were harmonized between the text outputs and PATSTAT by appending the application date and publication date to the patent identifier. To avoid mismatching documents between datasets all three identifiers were required to match in moving from text mined data to PATSTAT. In the case of the USPTO collection the direct match level was 96.8% and for the Patent Cooperation Treaty 99.2% indicating high resolution. In the case of the US collection the non-matching documents are primarily US Plant Patents (11,473 of 12,532 unmatched documents) that are not included in PATSTAT with the remainder arising from Statutory Inventions and non-matching document kind codes.

Patent Applicant Name Harmonization: Patent Applicant or Assignee names are noisy and may include multiple variations of the same name. To address this issue we used the ECOOM-EUROSTAT-EPO PATSTAT Person Augmented Table (EEE-PPAT 2012 edition) consisting of harmonized applicant names for use in PATSTAT. The methods used to develop EEE-PPAT are described in the following EUROSTAT documents:

1. Du Plessis, M., Van Looy, B., Song, X & Magerman, T. (2009) Data Production Methods for Harmonized Patent Indicators: Assignee sector allocation. EUROSTAT Working Paper and Studies, Luxembourg.
2. Magerman T, Grouwels J., Song X. & Van Looy B. (2009). Data Production Methods for Harmonized Patent Indicators: Patentee Name Harmonization. EUROSTAT Working Paper and Studies, Luxembourg.
3. Peeters B., Song X., Callaert J., Grouwels J., Van Looy B. (2009). Harmonizing harmonized patentee names: an exploratory assessment of top patentees. EUROSTAT working paper and Studies, Luxembourg.

The use of EEE-PPAT 2012 had a major impact on the harmonization of assignee names and respective patent rankings. However, we note two points. First, despite the major efforts at name harmonization described above further work is desirable. Based on the publications above we anticipate this will lead to an increase in the numbers of patent documents ascribed to an individual assignee. It may also lead to some minor variation in the top rankings.

A second, and separate, issue arises from mergers and acquisitions. The patent data in this article does not address mergers and acquisitions because of the difficulty of identifying reliable data sources and problems with subsequent demergers. In addition, a merger or acquisition does not necessarily involve a complete transfer of a patent portfolio. We presently know of no verified method to map transfers of patent ownership following mergers and acquisitions. We therefore limit our approach to including the name of an acquiring company in brackets after the original assignee (i.e. Pioneer Hi Bred is owned by Du Pont). We anticipate that detailed analysis of mergers and acquisitions would lead to greater concentration of patent ownership in areas such as agriculture.

The assignee data presented in Figure 1 is based on EEE-PPAT 2012. Discussion of top assignees in individual sectors is based on data retrieved from Thomson Innovation cleaned on the Patent Assignee Long Name based on matches with the INPADOC First Family member. This approach was adopted for the purpose of simplicity in discussing segments of data. Data on top assignees is indicative and confined to the core collections searched.

Cross Referencing with Thomson Innovation: As note above we used Thomson Innovation as an independent commercial data source for testing and validation of results. This data source has the advantage that 30,000+ records can be readily be retrieved for processing in Vantage Point. This proved to be particularly useful for investigating sub-sectors of activity. Harmonization was achieved using the publication numbers from the core index which, with very few exceptions, map directly into Thomson Innovation. In the case of a small number of sub-sectors assignee data presented in the supplementary tables is based on the cleaning of assignee data using the Patent Assignee (long) field with Thomson Innovation versions of the data. Name cleaning was performed by combining variant names of assignees based on a shared INPADOC first family member number. This method was used because our data does not presently permit easy filtering of assignees by International Patent Classification codes.

**b) Federation with Taxonomic Databases**: Federation was achieved with the Global Biodiversity Information Facility (GBIF) using GBIF data services and subsequent use of Catalogue of Life 2012 web services.

Federation with GBIF data allowed for the retrieval of kingdom information and available data on the geographic distribution of species (by country).

During this step we sought to resolve species names from the Global Names Index onto names within GBIF with a preference for accepted scientific names. Species names involving known synonyms were grouped onto the accepted name. In cases where no match was possible with an accepted scientific name the name was simply recorded in the Resolved Name field. Resolved Names feature accepted scientific names and cases where no match was recorded. The Names field is populated by variant names grouping onto the Resolved Names field based on data from GBIF.

In the course of calculating kingdom data it became clear that a number of species names were partial matches of full binomial names i.e. “*scherichia coli” should be “Escherichia coli”. We believe that partial matches are likely to arise from the OCR (optical character recognition) process for translating patent documents from their original portable document format (.pdf) into text files.

To resolve partial name matches we adopted a three step procedure. 1. Where a partial name could only match with one possible resolved name this was taken as a resolution. 2. Where a partial name could match to multiple possibilities the data was queried for co-occurrences. In the case of one co-occurrence this was taken as a valid match. 3. Where a match was unavailable from the above steps, we used the highest frequency match of a partial name match with a resolved name in the same documents. Partial name matches resolved using this procedure are listed in the Name field in Workbook S2, table S1.

Kingdom resolution: Our original data displayed a significant shortfall in species name matches to kingdom matches and led to the identification of the partial name match issue described above. In addressing this issue we identified additional full species names where no kingdom data had been retrieved. We developed a method to resolve this problem focusing on the genus name. However, because a genus name may validly appear in more than one kingdom (homonyms) a structured approach to matching was required that focused on: a) identifying genera with only one possible kingdom; b) identifying the available taxa in a genus within a candidate kingdom to correctly allocate the species. Resolution proceeded using the Catalogue of Life 2012 web service. Kingdom matches are provided in table S1. Species falling into the category unknown form a target for further work. Unresolved abbreviated names are excluded from the Unknown category in counts by kingdom presented in Figure 1.

Exceptions: At the time of research the classification of the species name *Vibrio cholerae* (cholera) was ambiguous in GBIF records and was listed under both Plantae and Bacteria. This species allocation was corrected based on manual review. The species *Variola major* (smallpox) is not presently listed in GBIF and does not appear in the index provided in Table S1. *Variola major* is included in the discussion on bioweapons/bioterrorism based on independent searches in Thomson Innovation.

Common Names & Major Food Crops: Our method focused on the identification of Latin species names in patent data based on the Global Names Index. The Global Names Index primarily includes Latin species names and known variants of Latin species names but also includes a smaller number of ‘common’ English names. To address this problem we used a stop list of common English terms to remove such names at the pattern generation step. Given the difficulty of predicting common name occurrences in the GNI list a small number of artefacts may remain in the final results.

The primary advantage of a search strategy using Latin names is the distinctiveness of the resulting strings in patent documents published in multiple languages. However, in some circumstances it is likely that a patent applicant will only use a common or non-taxonomic name (i.e. for a Chinese traditional medicine or a numeric identifier for a non-described organism in areas such as genomics). For the purpose of our research we assumed that a serious patent applicant will typically use a Latin species name. Future research could explore the use of common names but would require very significant investment and innovation in data cleaning methods.

The treatment of major food crops is an exception to the rule for common names. In separate work conducted for the International Treaty on Plant Genetic Resources for Food and Agriculture, we developed methods to generate patent statistics for the world’s major food crops. To test data capture using Latin species names we reviewed 47,183 patent claims from a set of 71,496 patent documents for agriculture identified using International Patent Classification codes. Comparison of the use of Latin species names and common names in the claims revealed major variance between data capture for Latin species names and common names. We therefore identified the English common names for the species concerned. We then confined the search to areas of technology involving plant genetic resources using International Patent Classification codes. This avoided the inclusion of common names in irrelevant areas of technology (i.e. rice cookers and potato fryers). In addition we confined the results to the Titles, Abstracts and Claims sections of patent documents. This combined approach has the effect of only including those common names where the documents are in some fundamental sense about these species. The method applied in the case of agricultural food crops is described in detail in Oldham & Hall (2013) Study 4: Intellectual Property, Informatics and Plant Genetic Resources, in Moeller, N. & Stannard, C. (eds.) (2013) *Identifying Benefit Flows: Studies on the Potential Monetary and Nonmonetary Benefits Arising from the International Treaty on Plant Genetic Resources for Food and Agriculture.* Rome: International Treaty on Plant Genetic Resources for Food and Agriculture of the Food and Agriculture Organization.

**Counting Patent Documents:**

We refer to a combination of publication counts and first filings. Publication counts are simple counts of the number of publications (i.e. applications and grants) that contain a species name. Counts of first filings are based on the International Patent Documentation Centre (INPADOC) first family member patent number from PATSTAT October 2011. Because patent documents are published more than once, commonly in more than one country, a system is required for grouping common documents together. This is provided by the INPADOC family system. Put simply an INPADOC patent family can be understood as a stack of documents published anywhere in the world that link back to a first or ‘priority‘ filing at the base of the stack. When we refer to first filings we refer to counts of documents at the bottom of the stack. We use the INPADOC system rather than other family definitions (i.e. Derwent) because INPADOC is the gold standard for patent statistics using PATSTAT.

Counting Patent Grants and Patent Applications: Trends in patent grants and applications were identified using patent kind codes. For the purpose of the analysis Kind Code A was taken to be an application and Kind Code B was taken to indicate a patent grant. Prior to 2001 US patents were only published when granted and could carry a Kind Code A. The data prior to 2001 was adjusted to reflect this by counting the records as grants. More accurate information on patent grants in Europe would be provided by investigating Legal Status data for additional European applications that became patent grants in member states of the European Patent Convention. This will be addressed in future research. Trends in grants and applications are therefore approximations based on accessible data.

Counting Species References by Section of Patent Document: Patent documents are divided into four main sections, the Title, Abstract, Description and Claims. We identified the occurrences of species names in each section of the documents. However, we would note that in some cases it is not readily possible to distinguish the demarcation between the Description and the Claims due to the variety of formats of patent documents. For this reason counts of the occurrences of species names in the claims are approximate.

**Identifying Technology Areas:**

To segment the data by technology area we used the 8^th^ edition of the International Patent Classification (IPC) of approximately 70,000 classification codes. Where a technology area is referred to in the article it is based on analysis of the IPC codes associated with the records for a particular species or group. The codes used to define technology areas discussed in the article are listed in the table below organized by the heading of the article section.

**Table C: International Patent Classification Codes for Technology Areas**

| Heading | IPC | Description (International Patent Classification Codes, 8^th^ Edition) |
| --- | --- | --- |
| Pharmaceuticals and Medicines | A61K31 | Medicinal preparations containing organic active ingredients |
| Pharmaceuticals and Medicines | A61K36 | Medicinal preparations of undetermined constitution containing material from algae, lichens, fungi or plants, or derivatives thereof, e.g. traditional herbal medicines |
| Pharmaceuticals and Medicines | A61P | Medical Or Veterinary Science; Hygiene - Therapeutic Activity Of Chemical Compounds Or Medicinal Preparations.  A61P3 Drugs for disorders of the metabolism,  see in particular, A61P3/04 - Anorexiants; Antiobesity agents, A61P3/10 for hyperglycaemia . e.g. antidiabetics  A61P13 - Drugs for disorders of the urinary system  A61P17 - Drugs for dermatological disorders  A61P25 - Drugs for disorders of the nervous system. See in particular, A61P25/22 Anxiolytics, A61P25/24 Antidepressants.  A61P31 - Antiinfectives i.e. antibiotics, antiseptics, chemotherapeutics. See in particular, A61P31/12 Antivirals  A61P33 - Antiparasitic agents  A61P35 - Antineoplastic agents |
| Cosmetics | A61K8, A61Q, | A61K8 - Cosmetics or Similar Toilet preparations;  A61Q - Specific use of Cosmetics or Similar Toilet Preparations |
| Genetic Engineering | C07H, C12N, C12Q, C12P | C07H - Sugars. Derivatives thereof; Nucleosides; Nucleotides; Nucleic Acids.  C12N **-** micro-organisms or enzymes; compositions thereof; propagating, preserving, or maintaining micro-organisms; mutation or genetic engineering; culture  C12Q - Measuring or testing processes involving enzymes or micro-organisms; compositions or test papers therefor; processes of preparing such compositions; condition-responsive control in microbiological or enzymological processes. Including C12Q1/68 for nucleic acids  C12P - fermentation or enzyme-using processes to synthesise a desired chemical compound or composition or to separate optical isomers from a racemic mixture |
| Biocides | A01N | A01N: Agriculture; Forestry; Animal Husbandry; Hunting; Trapping; Fishing -Preservation Of Bodies Of Humans Or Animals Or Plants Or Parts Thereof; Biocides, E.G. As Disinfectants, As Pesticides Or As Herbicides; Pest Repellents Or Attractants; Plant Growth Regulators |

**Table D: Patents Referenced by Subject Area and Species Name**

| Species | Patent Numbers | Detail |
| --- | --- | --- |
| **Country References** |  |  |
| *Boswellia serrata* | Note: 2009/2012 sample of 5 Patent Cooperation Treaty records from 190 records across the collections.  [WO2009010992A2](http://worldwide.espacenet.com/publicationDetails/biblio?DB=EPODOC&II=0&ND=3&adjacent=true&locale=en_EP&FT=D&date=20090122&CC=WO&NR=2009010992A2&KC=A2)  [WO2009031826A1](http://worldwide.espacenet.com/publicationDetails/biblio?FT=D&date=20090312&DB=EPODOC&locale=en_EP&CC=WO&NR=2009031826A1&KC=A1&ND=4)  [WO2009038875A1](http://worldwide.espacenet.com/publicationDetails/biblio?FT=D&date=20090326&DB=EPODOC&locale=en_EP&CC=WO&NR=2009038875A1&KC=A1&ND=4)  [WO2009045952A1](http://worldwide.espacenet.com/publicationDetails/biblio?FT=D&date=20090409&DB=EPODOC&locale=en_EP&CC=WO&NR=2009045952A1&KC=A1&ND=4)  [WO2009066303A2](http://worldwide.espacenet.com/publicationDetails/biblio?FT=D&date=20090528&DB=EPODOC&locale=en_EP&CC=WO&NR=2009066303A2&KC=A2&ND=4) | India |
| *Carpotroche brasiliensis* | [WO2009000055A1](http://worldwide.espacenet.com/publicationDetails/biblio?FT=D&date=20081231&DB=EPODOC&locale=en_EP&CC=WO&NR=2009000055A1&KC=A1&ND=4) | Brazil |
| *Lobostemon trigonus* | [US2007104728A1](http://worldwide.espacenet.com/publicationDetails/biblio?FT=D&date=20070510&DB=EPODOC&locale=en_EP&CC=US&NR=2007104728A1&KC=A1&ND=4)  [US2008063658A1](http://worldwide.espacenet.com/publicationDetails/biblio?FT=D&date=20080313&DB=EPODOC&locale=en_EP&CC=US&NR=2008063658A1&KC=A1&ND=4)  [US2008089946A1](http://worldwide.espacenet.com/publicationDetails/biblio?FT=D&date=20080417&DB=EPODOC&locale=en_EP&CC=US&NR=2008089946A1&KC=A1&ND=4)  [US7604823B2](http://worldwide.espacenet.com/publicationDetails/biblio?FT=D&date=20080313&DB=EPODOC&locale=en_EP&CC=US&NR=2008063658A1&KC=A1&ND=4)  [WO2007059441A2](http://worldwide.espacenet.com/publicationDetails/biblio?DB=EPODOC&II=0&ND=3&adjacent=true&locale=en_EP&FT=D&date=20070524&CC=WO&NR=2007059441A2&KC=A2) | South Africa |
| **Marine Species** |  |  |
| *Riftia pachyptila* | [US20030113747A1](http://worldwide.espacenet.com/publicationDetails/biblio?FT=D&date=20030619&DB=EPODOC&locale=en_EP&CC=US&NR=2003113747A1&KC=A1&ND=5) |  |
| *Beryx splendens* | [WO2005058476A1](http://worldwide.espacenet.com/publicationDetails/biblio?FT=D&date=20050630&DB=EPODOC&locale=en_EP&CC=WO&NR=2005058476A1&KC=A1&ND=4) |  |
| *Anoplopoma fimbria* | [WO2009034315A2](http://worldwide.espacenet.com/publicationDetails/biblio?FT=D&date=20090319&DB=EPODOC&locale=en_EP&CC=WO&NR=2009034315A2&KC=A2&ND=5) |  |
| *Bythograea thermydron* | [WO2004078962A1](http://worldwide.espacenet.com/publicationDetails/biblio?FT=D&date=20040916&DB=EPODOC&locale=en_EP&CC=WO&NR=2004078962A1&KC=A1&ND=4) |  |
| *Microstomus pacificus* | [WO2008148873A2](http://worldwide.espacenet.com/publicationDetails/biblio?FT=D&date=20081211&DB=EPODOC&locale=en_EP&CC=WO&NR=2008148873A2&KC=A2&ND=4) |  |
| **Antarctica** |  |  |
| *Pseudozyma antarctica* | [US7479381B1](http://v3.espacenet.com/textdoc?DB=EPODOC&IDX=US7479381) | Itaconic acid for use in paper and coatings |
| *Dissistichus mawsoni* | 1) [WO1991010361A1](http://v3.espacenet.com/textdoc?DB=EPODOC&IDX=WO1991010361)  2) [US5654279A](http://v3.espacenet.com/textdoc?DB=EPODOC&IDX=US5654279)  3) [WO2004022081A1](http://v3.espacenet.com/textdoc?DB=EPODOC&IDX=WO2004022081) | 1) Antifreeze proteins;  2) cryosurgery;  3) cosmetics |
| *Deschampsia antarctica* | 1) [WO2005049835A1](http://v3.espacenet.com/textdoc?DB=EPODOC&IDX=WO2005049835)  2) [WO2005049835A1](http://v3.espacenet.com/textdoc?DB=EPODOC&IDX=WO2008130701)  3) [WO2009064480A1](http://v3.espacenet.com/textdoc?DB=EPODOC&IDX=WO2009064480) | 1) Ice recrystallisation protein;  2) biofertilizer;  3) colon cancer |
| *Synoicum adareanum* | [WO2007035734A2](http://v3.espacenet.com/textdoc?DB=EPODOC&IDX=WO2007035734) | Cytotoxin compounds |
| *Aplidium cyaneum* | [WO2007054748A1](http://v3.espacenet.com/textdoc?DB=EPODOC&IDX=WO2007054748) | indole derivatives as anti-tumour compounds |
| *Chaenocephalus aceratus* | [WO2001009387A1](http://v3.espacenet.com/textdoc?DB=EPODOC&IDX=WO0109387) | hemapoietic genes |
| *Pagothenia borchgrevinki* | [WO2009136186A1](http://v3.espacenet.com/textdoc?DB=EPODOC&IDX=WO2009136186) | anti-icing proteins |
| *Euphausia superba* | [WO2008117062A1](http://v3.espacenet.com/textdoc?DB=EPODOC&IDX=WO2008117062) | supplements |
